# Intra-arterial administration of recombinant tissue-type plasminogen activator (rt-PA) causes more intracranial bleeding than does intravenous rt-PA in a transient rat middle cerebral artery occlusion model

**DOI:** 10.1186/2040-7378-3-10

**Published:** 2011-09-20

**Authors:** R Christian Crumrine, Victor J Marder, G McLeod Taylor, Joseph C LaManna, Constantinos P Tsipis, Philip Scuderi, Stephen R Petteway, Vikram Arora

**Affiliations:** 1 Research and Pre-clinical Development, Grifols Therapeutics, Inc., Research Triangle Park, North Carolina, USA; 2 Division of Hematology/Medical Oncology, Department of Medicine, David Geffen School of Medicine at UCLA, Los Angeles, CA, USA; 3 Department of Physiology and Biophysics, Case Western Reserve University, Cleveland, Ohio, USA

## Abstract

**Background:**

Intra-arterial (IA) administration of rt-PA for ischemic stroke has the potential for greater thrombolytic efficacy, especially for a large thrombus in the M1 or M2 segment of the middle cerebral artery (MCA). Intracranial hemorrhage (ICH) is a concern with IA or intravenous (IV) administration especially as the therapeutic window is extended. However, because IA administration delivers a higher local concentration of agent, the incidence and severity of ICH may be greater than with similar doses IV. We investigated the safety of rt-PA administration by IA compared to IV infusion following 6 hours of MCA occlusion (MCAo) with reflow in the spontaneously hypertensive rat (SHR).

**Methods:**

Male SHRs were subjected to 6 hours MCAo with 18 hours reflow using a snare ligature model. They were treated with IA saline, IA rt-PA (1, 5, 10, 30 mg/kg), or IV rt-PA (10 and 30 mg/kg) by a 10 to 60 minute infusion beginning approximately 1 minute before reflow. The rats were recovered for 24 hours after MCAo onset at which time Bleeding Score, infarct volume, and Modified Bederson Score were measured.

**Results:**

Greater hemorrhagic transformation occurred with 10 and 30 mg/kg rt-PA administered IA than IV. The IV 10 mg/kg rt-PA dosage induced significantly less bleeding than did the 1 or 5 mg/kg IA groups. No significant increase in infarct volume was observed after IA or IV treatment. Rats treated with 30 mg/kg rt-PA by either the IA or IV route had greater neurological dysfunction compared to all other groups.

**Conclusions:**

Administration of rt-PA by the IA route following 6 hours of MCAo results in greater ICH and worse functional recovery than comparable dosages IV. Significantly greater bleeding was observed when the IA dose was a tenth of the IV dose. The increased bleeding did not translate in larger infarct volumes.

## Background

Currently, the only approved pharmacological treatment for ischemic stroke is rt-PA via the intravenous (IV) route. However, recent experience with Intra-arterial (IA) infusion of rt-PA, alone or in combination with IV therapy or retrieval devices at the site of thrombus formation may provide a recanalization advantage over IV administration, especially in cases of high clot burden such as a thrombus located in the M1 or M2 segment of the middle cerebral artery [1-3]. IA administration of therapeutic agents within the first 3 hours of stroke symptom onset in the clinic is feasible [4,5] and is required for direct-acting fibrinolytic agents in development [6]. Although IA administration of a thrombolytic may improve the recanalization rate, such local infusion of agent may also increase the risk of intracerebral hemorrhage (ICH) [7] especially as the therapeutic window is extended [8]. Treatment with IV rtPA is effective when administered within 3 hours of stroke onset [9], but this narrow therapeutic window limits patient eligibility for thrombolytic therapy; estimated at only 1-2% [10]. Recently, the therapeutic window for IV r-tPA treatment was extended to 4.5 hours in Europe [11,12] and there is an ongoing clinical study aimed at further extending this window to 6 hours [13].

The safety of IA rt-PA administration following extended ischemic durations with respect to IV administration has not been heretofore evaluated. In this report, we compare IA- versus IV-delivered rt-PA in a pre-clinical model in rats that mimics the clinical situation.

## Methods

### Animals

Adult male spontaneously hypertensive rats (SHR) weighing 330 - 380 g were purchased from the Charles River Research facilities, Raleigh, NC. Animal experiments were conducted at facilities located at the North Carolina State University (NCSU) College of Agriculture and Life Sciences (CALS). The animal use protocol was reviewed and approved by the NCSU IACUC prior to the initiation of the studies and was performed in compliance with standards set forth by the National Research Council publication, *Guide for the Care and Use of Laboratory Animals*.

Upon arrival, the rats were assigned a number by the animal facility staff and housed individually. They were allowed at least one week to acclimate. The rats were on a 12 hour light/dark diurnal cycle and food and water were provided *ad libitum*. To conserve test article, rats were assigned to experimental groups in tandem (2 rats to the same group/experimental day) as outlined in Table 1.

**Table 1:** Experimental groups and dosing details

Group	n	Dose	[Test Article]	Injection Volume	Infusion Duration
Saline	5	Dose Vol	---	1 μL/g BW	10 min

Vehicle	5	Dose Vol	---	1 μL/g BW	10 min

rt-PA, IA Infusion	5	1 mg/kg	1 mg/mL	1 μL/g BW	10 min
	
	7	5 mg/kg	5 mg/mL	1 μL/g BW	10 min
	
	5	10 mg/kg	5 mg/mL	2 μL/g BW	20 min
	
	5	30 mg/kg	5 mg/mL	6 μL/g BW	60 min

rt-PA, IV Infusion	5	10 mg/kg	5 mg/mL	2 μL/g BW	20 min
	
	5	30 mg/kg	5 mg/mL	6 μL/g BW	60 min

The infusion rate of the test article depended on the body weight (BW) of the rat. The total volumes infused averaged 0.35 mL, 0.7 mL and 1.95 mL for infusion durations of 10, 20 and 60 minutes, respectively.

### General animal preparation

All rats were fasted overnight to provide a low consistent plasma glucose concentration before surgery. The rats were anesthetized with isoflurane (5% in O_2_) in an induction chamber and orotracheally intubated. For pain relief, all rats received 0.03 mg/kg buprenorphine, subQ (PharmaForce, Inc., Columbus, Ohio). Anesthesia was maintained by spontaneous ventilation of isoflurane (1.5-3% in O_2_). The ventral tail artery was cannulated (RPT-037, RenaPulse tubing, Braintree Scientific, Inc., Braintree MA or PE50 tubing) by cutdown. The arterial line was used to continuously monitor blood pressure and heart rate (Ponemah Physiological Monitoring System, DSI, Cleveland, Ohio) and to obtain arterial blood for blood gas determinations (i-Stat, Heska AG, Switzerland). Following arterial catheterization, the rats were artificially ventilated to maintain PaCO_2 _between 35 and 45 mmHg. Continued isoflurane anesthesia (1-1.5%) was driven by compressed air supplemented with O_2 _to maintain PaO_2 _between 95 to 170 mmHg. Body temperature was maintained at or slightly above 37°C using a heat lamp in a feedback circuit with a rectal temperature probe (TCAT-2 Temperature Controller, Physitemp Instruments, Inc., Clifton, NJ). Just before MCAo, reflow, and during the blood flow studies, the isoflurane was reduced to approximately 1% (from 1.5%) to allow for normalization of blood pressure while maintaining an adequate level of anesthesia.

### Blood flow measurements

Rats were placed in dorsal recumbency and the common carotid (CCA), external carotid (ECA), internal carotid (ICA), occipital and pterygopalatine arteries were isolated by cutdown. A perivascular blood flow probe (Transonic Systems Inc., Ithaca, NY) was placed around the CCA for continuous blood flow monitoring. Sequentially, the occipital, the external carotid, and the pterygopalatine arteries were occluded leaving the blood flow to the brain via the extra-cranial internal carotid artery (EC-ICA).

### Transient middle cerebral artery occlusion (MCAo) model

**Figure 1:**
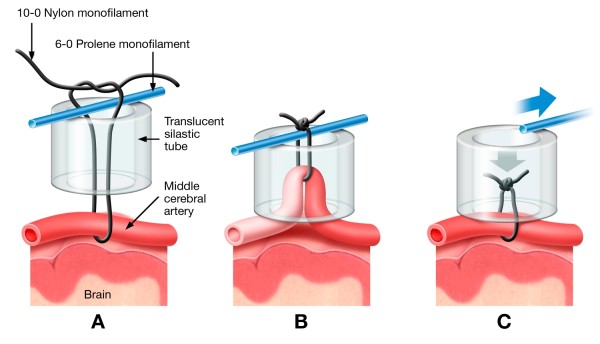
**Snare ligature MCAo model**. (**A**) Construction of the snare ligature. (**B**) Occlusion of the MCA. (**C**) Reflow of the MCA.

Six (6) hours of transient MCAo was induced in the SHR using the snare ligature model first described in mice [14], adapted to the rat [15] and illustrated in Figure 1. The head was immobilized in a specially designed head holder and a skin incision was made between the eye and the external auditory canal. Using a surgical microscope (Zeiss OPMI-6C, Prescott's Inc., Monument, CO), the temporalis muscle was bisected and retracted and a small craniotomy was performed using a dental drill anteromedial to the zygomatic arch-squamosal bone junction. The dura was opened and the MCA was isolated from the arachnoid and pia by blunt dissection proximal to the inferior cerebral vein. The snare ligature was constructed as illustrated in Figure 1A using a strand of 10-0 nylon suture, a piece of 6-0 prolene monofilament and silastic tubing (ID 0.76 mm × OD 1.65 mm × ~1 mm h; Dow Corning Corporation). The MCA was gently pulled into the lumen of the silastic tubing resulting in occlusion of the artery (Figure 1B). MCAo was visually confirmed through the surgical microscope (40 × magnification). A piece of Gelfoam^® ^was placed in the cranial deficit and the surgical site was closed in two layers. The rat was recovered from anesthesia, extubated and returned to a clean cage. The normal bedding in the cage was replaced with an absorbent cage pad (Recovery Pad ™, Bed O' Cobs/The Andersons, Distributor: Granville Milling Co., Creedmoor, NC). Between the MCAo surgery and the removal of the snare ligature, the rats had free access to gel style food (Hydro Gel and Diet Gel-Recovery; H2O, Portland, Maine). The location of the snare ligature, distal to the lenticulostrate arteries but proximal to the inferior cerebral vein, resulted in nearly pure cortical infarction (Additional file 1, Figure S1).

We chose the rat snare ligature model of MCAo to strictly control the ischemic duration which is not possible in a thromboembolic model. Further, in a thromboembolic model, control groups such as saline and vehicle become de facto permanent occlusion animals and thus a proper control for specific ischemic durations with reflow is not practicable. In addition, the snare ligature model allows for the initiation of local IA dosing to immediately precede reflow. Thus, the drug would be in high concentration during the first pass of blood into the ischemic tissue thereby better mimicking human thrombolytic treatment by IA administration. This is not possible using an intraluminal model where a significant delay between reflow and IA dosing would occur due to exchanging the occluding filament for the dosing catheter. This is in addition to other complications associated with the intraluminal model such as pre-mature reflow [16], ischemia to the hypothalamus resulting in hyperthermia [17], distension of the MCA (personal observations) and the possibility of filament related subarachnoid hemorrhage [16].

### rt-PA dosing solution preparation

Lyophilized rt-PA (Alteplase, recombinant, Genentech, South San Francisco, CA, USA) was solubilized at an initial concentration of 5 mg/mL using the provided diluent (Water for Injection, USP (WFI) and further diluted as needed to 1 mg/mL using saline for injection. Unused portions (1 and 5 mg/mL) were divided into 1-3 mL aliquots, snap frozen and stored at -80°C [18].

**Figure 2:**
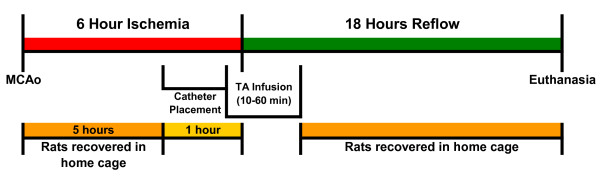
**Experimental timeline**.

Concentrations of rt-PA higher than 5 mg/mL were not feasible because of osmolality (the 5 mg/mL solution was 793 mOsmol/L) and solubility limitations (particularly important for IA administration). The rate of test article infusion IA or IV was 0.033-0.038 mL/min. The experimental groups and dosing details are provided in Table 1. A timeline of the experiment is provided in Figure 2.

### rt-PA dose administration

#### IA dosing

**Figure 3:**
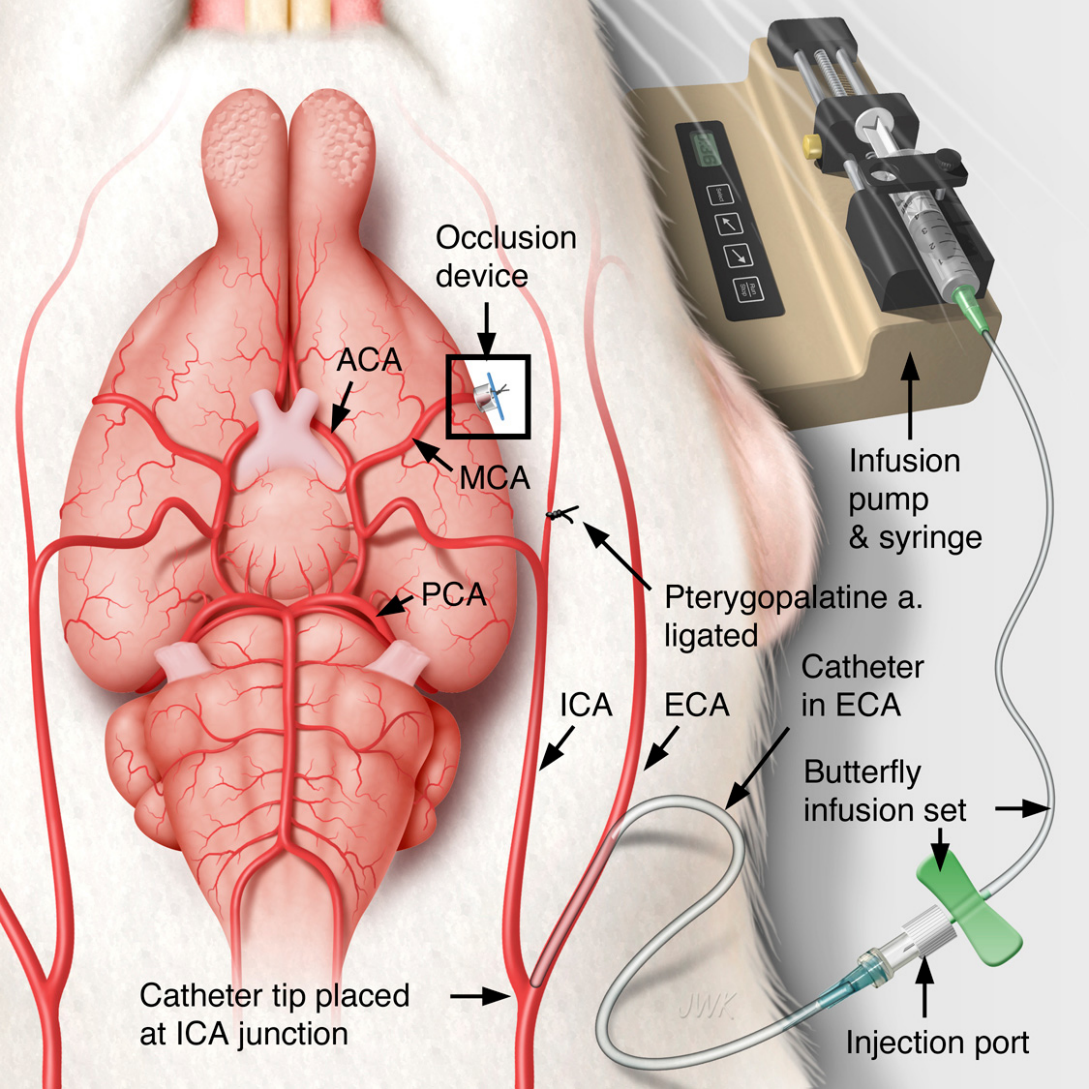
**Schematic of IA dosing**. For clarity, the illustration shows a ventral analogy. In practice, once the extra-cranial internal carotid artery (EC-ICA) was cannulated, the rat was gently rolled onto his right flank and the injection port was secured to the left flank. The head was, again, immobilized in the head holder. During this process, patency of the catheter was maintained by saline infusion (0.75 mL/hr). Infusion of test article was accomplished by replacing the saline flush syringe butterfly assembly with an assembly containing the test article. The infusion of the test article was initiated approximately 1 minute before dismantling the snare ligature while the head was secured in the head holder.

The IA dosing was accomplished by a catheter in the EC-ICA as illustrated in Figure 3. Approximately 5 hours after MCAo, the rats were reanesthetized, intubated and artificially ventilated as previously described. The CCA, ECA, ICA and pterygopalatine arteries were isolated by cutdown. The ECA and pterygopalatine arteries were ligated and a catheter was introduced into the ECA and advanced to the ICA and CCA juncture. The catheter was secured to the ECA and the rat was gently rolled onto his right flank and the injection port of the catheter assembly was sutured to the left flank. The head of the rat was immobilized as previously described and the snare ligature was exposed. The test article infusion was initiated and approximately 1 minute later, the snare ligature was dismantled by pulling out the 6-0 prolene, removing the silastic tubing and cutting and removing the 10-0 suture (Figure 1C). The surgical site was flooded with papaverine to dilate the MCA. Reflow was confirmed by visual inspection through the surgical microscope. Following dosing, the EC-ICA catheter was removed, the surgical sites were closed and the rat was returned to his home cage.

The IA dosing catheter was constructed from a PTFE sub-lite wall tubing (SUBL-160, OD/ID 0.41/0.25 mm; Braintree Scientific, Inc., Braintree, MA) attached to a 30 g needle which was attached to an injection port (Additional file 1, Figure S2). To maintain patency, a saline infusion (0.75 mL/hr) was administered as illustrated in Figure 3. The saline infusion was begun before placing the catheter into the ECA to ensure no air was infused into the ICA during the catheterization procedure. For dosing, the saline syringe/butterfly infusion set assembly was replaced with an assembly containing the test article and the infusion pump was adjusted to the proper rate.

Table 2 shows the blood flow in the CCA, ECA, ICA, occipital and pterygopalatine arteries in the normal SHR. The blood flow in the EC-ICA with all other arteries occluded was approximately 0.89 mL/min. Thus, the test article infusion comprised approximately 4% of the ICA blood flow to the brain. For the 10 and 30 mg/kg doses, the infusion rate remained the same but the infusion duration was extended from 10 minutes to 20 and 60 minutes, respectively (See Table 1).

**Table 2:** Blood flow for the indicated arteries

Artery	Blood Flow (mL/min)	% EC-ICA Blood Flow*
Common Carotid Artery	3.49 ± 0.19	----

Occipital Artery	0.43 ± 0.09	----

External Carotid Artery	1.22 ± 0.28	----

Pterygopalatine Artery	1.52 ± 0.08	63%

Intra-Cranial Internal Carotid Artery (blood flow to the brain)	0.89 ± 0.04	37%

The arteries were isolated by cutdown and a perivascular blood flow probe was placed around the common carotid artery. Arteries were then sequentially occluded and blood flow for each artery was determined by subtraction. * Percent of the extra-cranial internal carotid artery (EC-ICA) blood flow delivered to these arteries (the rat EC-ICA bifurcates just before entering the skull). The values are the mean ± SEM (n = 4).

#### IV dosing

rt-PA was administered through a catheter, placed by cutdown, in the external jugular vein. Following dosing, the catheter was removed and the surgical sites closed. The rats were recovered from anesthesia and returned to their home cage. All rats received a second dose of buprenorphine (0.03 mg/kg, subQ) during recovery from anesthesia following the completion of dosing.

Ten (10) mg/kg IV rt-PA is considered the efficacious dose for thrombolysis in the rat whereas the human dose is 0.9 mg/kg. The 10-fold discrepancy is based on an *in vitro *clot lysis experiment showing that rat whole blood clots are 10 times less sensitive to human rt-PA [19]. Recent *in vivo *experiments tend to support that conclusion [20,21].

### Infarct volume analysis

Twenty four hours after onset of MCAo, the rats were deeply anesthetized with isoflurane (5% in O_2_) and the brain was perfused *in situ *for approximately 90 seconds with heparinized saline (10 U/mL). The brain was removed, placed in ice cold saline and examined for gross superficial hemorrhage. Digital photographs of the brain surface were obtained.

Sequential coronal sections were obtained every 2 mm throughout the neocortex (8-9 sections/brain). The sections were stained with 1-2% 2, 3, 5 triphenyl tetrazolium chloride (TTC) [22] at 37°C in the dark to effect (~15 min incubation time). The sections were fixed in 10% buffered formalin for 24 hours. Each fixed brain section was digitally photographed with a ruled standard.

The digital photographs of the fixed TTC stained brain sections were imported into an image analysis program (Image-Pro Plus v4.5, Media Cybernetics, Inc., Bethesda, MD) for infarct volume measurement. The infarct volume, reported in mm^3^, was determined by the indirect method.

### Bleeding score

**Figure 4:**
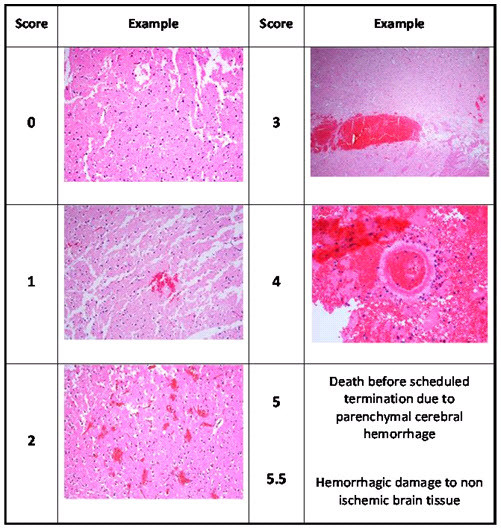
**Examples of the bleeding severity score**. Two 10 μm sections were obtained from each 2 mm TTC stained section and stained with H&E.

Two complete 10 μm sections, spaced 250 μm apart, were obtained from each original 2 mm TTC stained section and processed for H&E histopathology (16-18; 10 μm sections/rat brain). Each section was evaluated for Bleeding Score, according to the following definitions (Examples are shown in Figure 4):

0 = Normal ischemic damage - no hemorrhage

1 = Dispersed individual petechiae

2 = Confluent petechiae

3 = Hemorrhagic infarction

4 = Large cerebral hemorrhage

5 = Animal found dead due to ICH before planned termination

5.5 = Hemorrhage to non ischemic brain tissue

The overall score for the rat brain was the highest score of the evaluated sections.

### Neurological function analysis

The rats were assessed for neurological function twice during the study, once prior to the induction of anesthesia to place the catheter for test article administration and again just before euthanasia. The first assessment was to screen for stroke presence; any rat not displaying a score of at least 2 was eliminated from the study. The second assessment was performed just before euthanasia to determine the final functional recovery of the rat; only the second score was analyzed as an experimental variable.

The rats were assessed for neurological function using a Modified Bederson Score [23] in an unblinded manner by RCC and GMT with the following definitions:

Score 0: No apparent neurological deficits

Score 1: Body torsion present

Score 2: Body torsion with right side weakness

Score 3: Body torsion, right side weakness with circling behavior

Score 4: Unresponsiveness - euthanasia < 3 hours after dosing

Score 5. Seizure Activity - immediate euthanasia or death before scheduled termination due to ICH.

### Exclusion/Inclusion criteria

Animals were excluded from the study if one of the following occurred: breakage of the MCA during occlusion or recanalization; breakage of a side branch of the MCA causing bleeding at the surgical site; Modified Bederson Score less than 2 prior to start of the reflow surgery; reflow cannot be visually confirmed; excessive bruising of the brain during occlusion surgery; observation of air bubbles or particles in the ICA catheter during infusions; surgical complications requiring euthanasia, or a plasma glucose concentration > 200 mg/dL prior to MCAo.

### Statistics

Statistical analysis of the infarct volume and physiological variables between the experimental groups was accomplished by ANOVA followed by Tukey-Kramer HSD multiple comparison test (JMP statistical software, SAS Institute, Inc., Cary, NC).

Statistical analysis of the Bleeding Score and the Modified Bederson Score data was performed using the Kruskal-Wallis one way ANOVA test for non-parametric data followed by Newman-Keuls multiple comparison test using the GBstat statistical package (Dynamic Microsystem, Inc., Silver Spring, MD).

Analysis of covariance (ANCOVA) with the dependent variables of infarct volume, Bleeding Score and Modified Bederson Score, respectively, with the independent variables of dose and route was performed using SAS^® ^software version 9.2 (SAS Institute, Inc. Cary, NC).

To determine the number of rats in each group, a power analysis of the vehicle group from a contemporary pilot study was performed using infarct volume as the test variable. To observe a statistically significant difference of 30% between the means of the experimental groups at an α level of 0.05 and a β level of 0.8, an n of at least 5 animals would be required. This is consistent with Brint et al [24] and with our past experience using the SHR in stroke studies. All of the data are presented as mean ± SEM.

The infarct volume and Bleeding Score were determined in a blinded fashion by CPT in the laboratory of JCL (Case Western Reserve University, Cleveland, Ohio).

### Preliminary control studies

#### Temporal infarct progression

Historical unpublished temporal progression data generated by two of the authors (RCC and JCL) at Case Western Reserve University in Cleveland, Ohio (1987) suggests that 6 hours of MCAo with reflow should result in maximal large stable infarct volumes (Additional file 1, Figure S3). These data are similar to those of Aronowski et al [25] and are consistent with the "time is brain" concept in the clinic [26-28]. We chose a 6 hour ischemia duration to better mimic extended symptom onset to treatment delay in the clinic and we chose the spontaneously hypertensive rat (SHR) because of the reproducibility of the ischemic lesion.

#### Sham animals

To determine the contribution of the surgical procedure and EC-ICA saline infusion on infarct volume, two sham groups were performed. In the first group (surgical shams, n = 3), the MCAo surgical procedure was performed, the artery was elevated into the silastic tube (not occluded) for less than 5 seconds and then the snare ligature was immediately dismantled. In the second group (ICA sham, n = 2), rats were exposed to the surgical sham procedure followed by saline infusion into the EC-ICA 6 hours later. No visible damage to the brain was apparent by gross inspection of the brain or in TTC stained or in H&E stained histological sections (Additional file 1, Figures S4 and S5).

#### Reflow confirmation after 6 hours MCAo

To confirm reflow of the MCA following removal of the snare ligature, 2 rats were subjected to 6 hours MCAo, the EC-ICA was cannulated and within 5 minutes following removal of the snare ligature, pink latex was infused into the cerebral circulation through the EC-ICA catheter. In both rats, the latex demarked the MCA vascular tree distal to the occlusion site, confirming reflow (Additional file 1, Figure S6).

## Results

### Exclusion of animals

MCAo surgery was performed on 56 rats, of which 42 were assigned to the different experimental groups. Exclusions were for breakage of the MCA or a side branch (3 rats), bleeding complications prior to craniotomy (3 rats), bruising of brain tissue (2 rats), occlusion device malfunction (2 rats), clot in the ICA just distal to the pterygopalatine artery bifurcation (1 rat) and technical difficulties (3 rats).

### Physiological variables

All of the physiological variables were within normal range throughout the MCAo and reflow surgeries (Table 3). In all groups, the plasma glucose values prior to MCAo were significantly lower than prior to reflow; the former values reflect fasting the rats overnight and the latter values indicate that the rats were eating the gel style food between the two surgeries.

**Table 3:** Physiological variables

Pre Ischemia	Saline IA (n = 5)	rt-PA: 1 mg/kg IA (n = 5)	rt-PA: 5 mg/kg IA (n = 7)	rt-PA: 10 mg/kg IA (n = 5)	rt-PA: 30 mg/kg IA (n = 5)	rt-PA: 10 mg/kg IV (n = 5)	rt-PA: 30 mg/kg IV (n = 5)
pH	7.43 ± 0.03	7.48 ± 0.01	7.49 ± 0.01	7.47 ± 0.02	7.46 ± 0.03	7.51 ± 0.02	7.44 ± 0.01

PaCO_2 _(mmHg)	39.1 ± 4.5	36.2 ± 1.0	36.2 ± 1.2	38.7 ± 1.2	38.3 ± 2.5	35.2 ± 2.1	41.3 ± 1.6

PaO_2 _(mmHg)	99.2 ± 6.5	118.0 ± 6.0	111.9 ± 4.0	164.6 ± 7.5	109.0 ± 8.7	110.0 ± 11.9	106.4 ± 4.5

Glu (mg/dL)	138 ± 9	137 ± 4	131 ± 8	152 ± 10	136 ± 13	139 ± 6	136 ± 13

MABP (mmHg)	116 ± 17	105 ± 7	145 ± 12	119 ± 13	148 ± 15	132 ± 10	141 ± 15

HR (beats/min)	362 ± 10	353 ± 6	332 ± 6	351 ± 12	358 ± 11	347 ± 16	334 ± 40

Hct (%PCV)	43.8 ± 1.9	45.6 ± 0.7	44.6 ± 0.6	45.6 ± 0.5	44.6 ± 0.7	45.2 ± 0.5	45.2 ± 0.5

Body Temp (°C)	37.1 ± 0.1	37.0 ± 0.0	37.1 ± 0.1	37.0 ± 0.0	37.1 ± 0.1	37.0 ± 0.0	37.1 ± 0.0

Body Wt (g)	329.0 ± 7.0	328.4 ± 6.5	345.4 ± 3.2	339.6 ± 12.7	318.2 ± 7.8	328.6 ± 14.3	330.8 ± 2.0

**Pre Reflow**							

pH	7.47 ± 0.03	7.42 ± 0.02	7.48 ± 0.02	7.44 ± 0.01	7.45 ± 0.02	7.42 ± 0.04	7.45 ± 0.02

PaCO_2 _(mmHg)	34.6 ± 2.7	38.7 ± 2.0	36.6 ± 1.2	38.1 ± 1.2	37.1 ± 1.0	38.6 ± 2.6	38.9 ± 1.6

PaO_2 _(mmHg)	157.4 ± 2.7	167.4 ± 17.6	167.1 ± 10.2	164.6 ± 7.5	149.4 ± 12.3	145.8 ± 13.4	136.0 ± 6.6

Glu (mg/dL)	195 ± 38	348 ± 63	299 ± 49	379 ± 81	196 ± 13	243 ± 44	204 ± 11

MABP (mmHg)	145 ± 18	138 ± 16	117 ± 14	145 ± 12	137 ± 14	138 ± 19	164 ± 16

HR (beats/min)	377 ± 7	370 ± 9	347 ± 9	367 ± 7	368 ± 9	364 ± 16	340 ± 34

Hct (%PCV)	43.4 ± 2.6	46.0 ± 1.0	45.3 ± 0.7	46.4 ± 1.1	42.6 ± 1.0	45.6 ± 0.8	45.6 ± 1.0

Body Temp (°C)	37.1 ± 0.1	37.1 ± 0.0	37.0 ± 0.1	37.1 ± 0.0	37.0 ± 0.1	37.6 ± 0.4	37.0 ± 0.1

Rats were subjected to MCAo with reflow as described. Blood from the tail arterial line was obtained and analyzed for blood gas values within 5 minutes of MCAo and of removal of the snare ligature (pre-reflow values). The values are the mean ± SEM.

### Bleeding score

**Figure 5:**
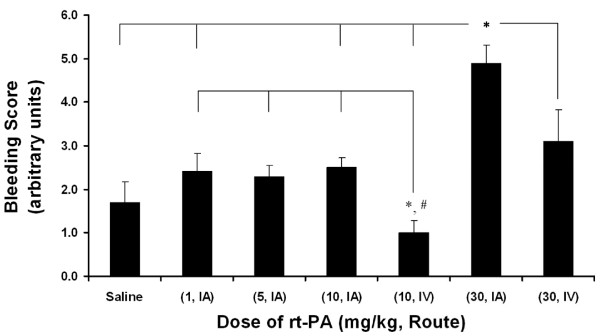
**Bleeding score**. Rats were subjected to 6 hours MCAo followed by approximately 18 hours of reflow. Treatment with rt-PA began 1 minute before reflow. The values are the mean ± SEM (n = 5, 5, 7, 5, 5, 5, 5, respectively). * p < 0.05 compared to indicated groups (Kruskal-Wallis, Newman-Keuls). # p < 0.05 compared to 30 mg/kg rt-PA, IV (Kruskal-Wallis, Newman-Keuls).

**Figure 6:**
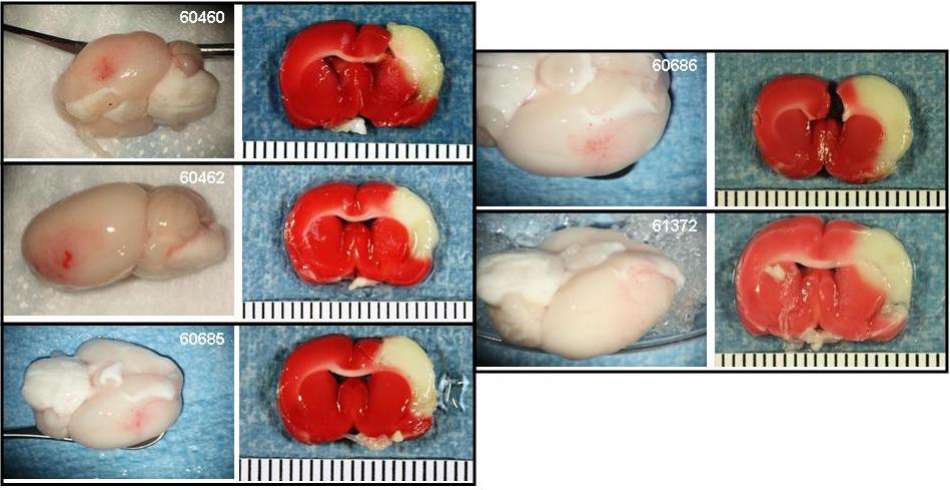
**IA saline control animals**. Rats were subjected to 6 hours MCAo with reflow. Treatment with IA saline commenced 1 minute before reflow. Photographs of the gross brain and a TTC stained coronal section from each animal in the group are displayed.

**Figure 7:**
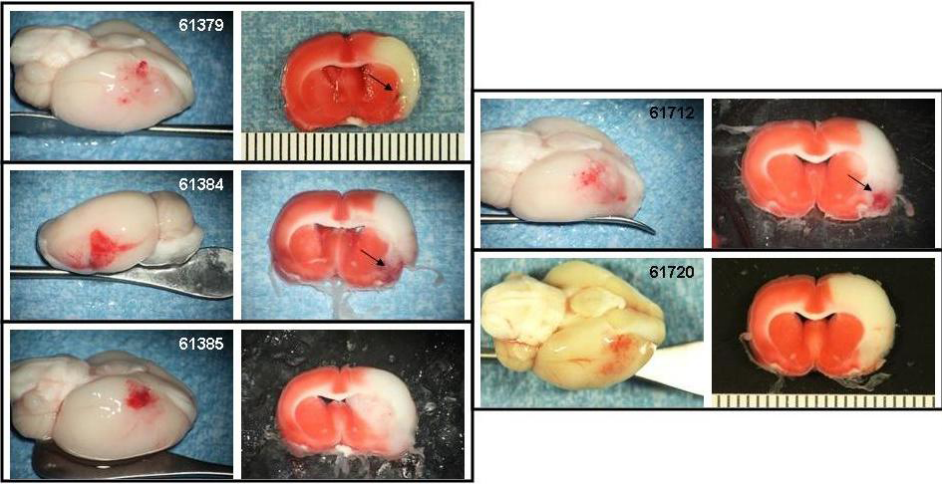
**IA rt-PA 10 mg/kg animals**. Rats were subjected to 6 hours MCAo with reflow. Treatment with IA rt-PA 10 mg/kg commenced 1 minute before reflow. Photographs of the gross brain and a TTC stained coronal section from each animal in the group are displayed. Arrows indicate hemorrhage.

**Figure 8:**
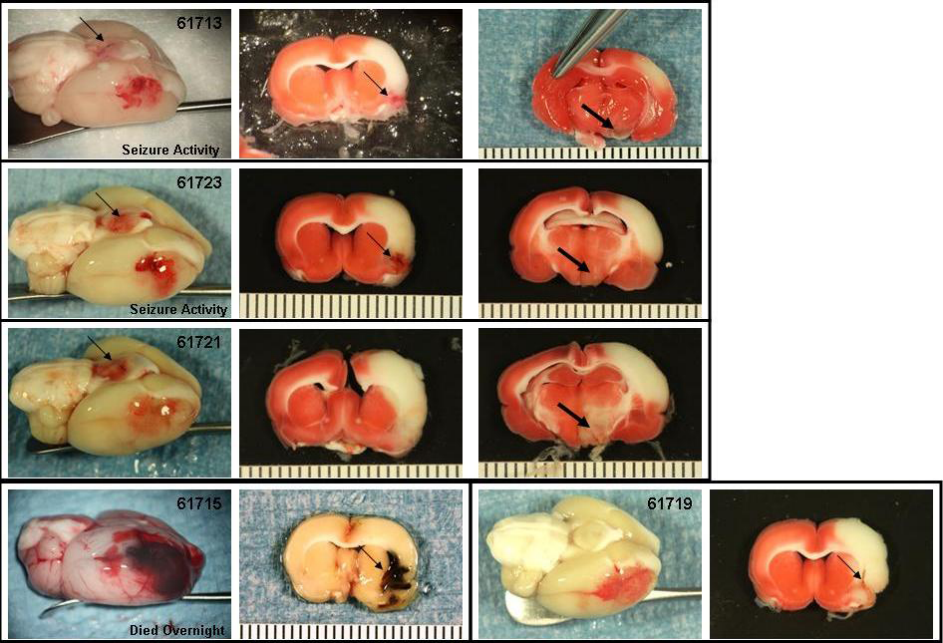
**IA rt-PA 30 mg/kg animals**. Rats were subjected to 6 hours MCAo with reflow. Treatment with IA rt-PA 30 mg/kg commenced 1 minute before reflow. Photographs of the gross brain and a TTC stained coronal section from each animal in the group are displayed. Thin arrows indicate hemorrhage. Thick arrows indicate damage to the hypothalamus.

**Figure 9:**
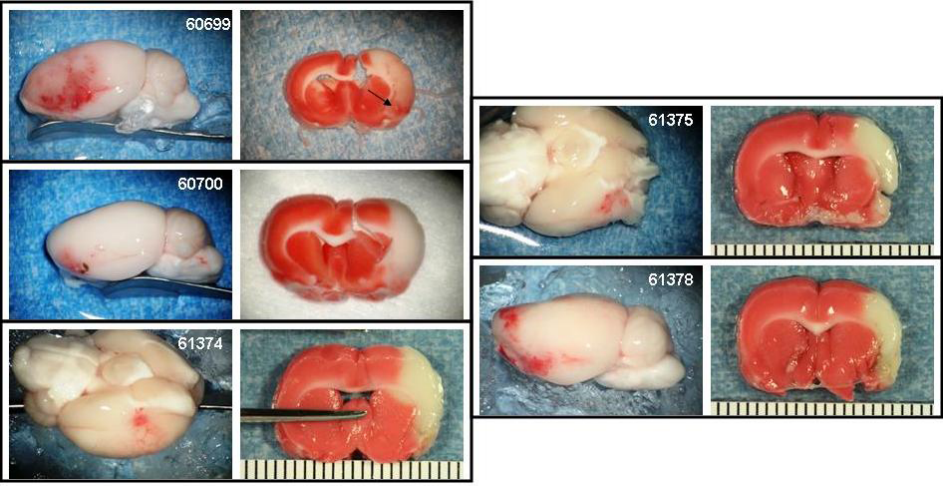
**IV rt-PA 10 mg/kg animals**. Rats were subjected to 6 hours MCAo with reflow. Treatment with IV rt-PA 10 mg/kg commenced 1 minute before reflow. Photographs of the gross brain and a TTC stained coronal section from each animal in the group are displayed. Arrows indicate hemorrhage.

**Figure 10:**
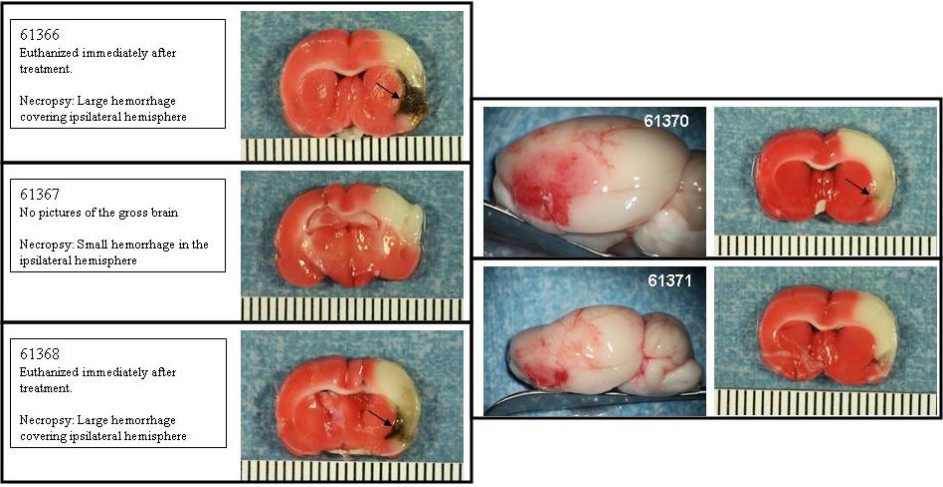
**IV rt-PA 30 mg/kg animals**. Rats were subjected to 6 hours MCAo with reflow. Treatment with IV rt-PA 30 mg/kg commenced 1 minute before reflow. Photographs of the gross brain and a TTC stained coronal section from each animal in the group are displayed. Arrows indicate hemorrhage.

The Bleeding Score for the experimental groups are shown in Figure 5. Photographs of the gross brain and a TTC stained section of the brain at the level of the basal ganglia and in some cases, at the level of the anterior hippocampus are presented for the animals treated with saline (Figure 6), 10 or 30 mg/kg rt-PA IA (Figures 7 and 8, respectively), and 10 or 30 mg/kg IV (Figures 9 and 10, respectively).

The Bleeding Severity Score of the saline group (1.7 ± 0.5) and the groups treated IA with 1, 5 and 10 mg/kg rtPA were low (2.2 ± 0.4, 2.4 ± 0.3 and 2.5 ± 0.2, respectively) and similar (Figures 5, 6 and 7). Markedly more severe bleeding was observed with 30 mg/kg rtPA delivered IA (4.9 ± 0.4) compared with all other groups except 5 mg/kg IA rt-PA. One rat treated with 30 mg/kg rt-PA IA had a massive space-occupying hemorrhage and did not survive to the scheduled end of the experiment (# 61715, Figure 8). Three rats in this group had left brain stem hemorrhage (# 61713, 61723, and 61721 Figure 8, left panels, respectively; thin arrows) resulting in hypothalamic damage (Figure 8, right panels, respectively; thick arrows). Two of these rats developed seizure activity after dosing that necessitated euthanasia.

An increase in bleeding score occurred with increased doses of rtPA IV (Figure 5). The 30 mg/kg IV rtPA dose showed a higher Bleeding Score (3.1 ± 0.7) as compared to the 10 mg/kg rtPA IV dose (1.0 ± 0.3), and space-occupying hemorrhage occurred in 2 of 5 rats (61366 and 61368, Figure 10; right panel, respectively; thin arrows). Four of 5 rats were euthanized approximately 3 hours after terminating anesthesia after agent administration because of unresponsiveness. None of the rats in this group had brain stem hemorrhage, seizure activity or hypothalamic damage.

The 10 mg/kg IA rt-PA dose caused significantly more bleeding than did 10 mg/kg IV (Figures 5, 7 and 9). Indeed, rats that received only 1 or 5 mg/kg IA rt-PA had significantly more bleeding than rats receiving 10 mg/kg rt-PA IV. Similarly, 30 mg/kg IA rt-PA caused significantly more bleeding than did 30 mg/kg IV (Figures 5, 8 and 10). The Bleeding Score in rats receiving 30 mg/kg rt-PA IV was not significantly different that in rats receiving 10 mg/kg rt-PA IA. Multivariate analysis revealed a statistically significant effect of dose and route on Bleeding Score (Table 4).

**Table 4:** Analysis of Covariance (ANCOVA)

Dependent Variable	Independent Variable	p-Value
Bleeding Score	Dose	< 0.0001*

	Route	< 0.0001*


Infarct Volume	Dose	0.1787

	Route	0.2020


Modified Bederson Score	Dose	< 0.0001*

	Route	0.0310*

Rats were subject to 6 hours of MCAo and then treated with saline or rt-PA. The indicated dependent variables were analyzed in relation to the independent variables.

### Infarct volume

**Figure 11:**
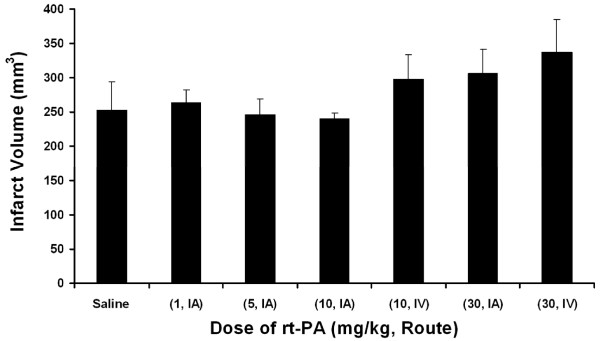
**Infarct volume**. Rats were subjected to 6 hours MCAo followed by approximately 18 hours of reflow. Treatment with rt-PA began 1 minute before reflow. The values are the mean ± SEM (n = 5, 5, 7, 5, 5, 5, 5, respectively). No significant differences were found (ANOVA).

The infarct volume after 6 hours of ligature ischemia followed by 18 hours reflow was not significantly different for all treatments (Figure 11), although there was a trend towards a larger infarct volume using the highest dose (30 mg/kg) of rt-PA either IA or IV. Saline treatment (252 ± 41 mm^3^) and treatment with 1 mg/kg IA (264 ± 19 mm^3^), 5 mg/kg IA (247 ± 23 mm^3^) and 10 mg/kg IA (240 ± 8 mm^3^) had very similar infarct volumes, whereas infarct volume after treatment with 30 mg/kg rtPA IA tended to be larger (306 ± 35 mm^3^). Infarct volumes after IV rtPA at 10 and 30 mg/kg (298 ± 37 and 338 ± 48 mm^3^, respectively) tended to be larger than saline control. Multivariate analysis showed no statically significant effects of dose or route on infarct volume (Table 4).

### Modified Bederson score

**Figure 12:**
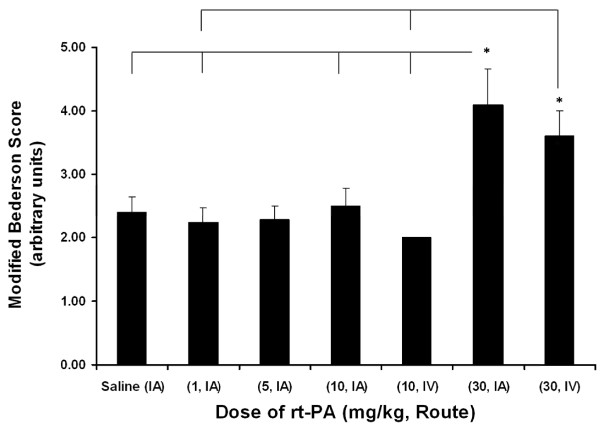
**Neurological function score**. Rats were subjected to 6 hours MCAo followed by approximately 18 hours of reflow. Treatment with rt-PA began 1 minute before reflow. The values are the mean ± SEM (n = 5, 5, 7, 5, 5, 5, 5, respectively). * p < 0.05 compared to indicated groups (Kruskal-Wallis, Newman-Keuls)

The Modified Bederson Score, reflecting neurological function 24 hours following MCAO (Figure 12) was nearly identical in rats treated with saline (2.4 ± 0.24), with IA rt-PA at 1 mg/kg (2.25 ± 0.22), 5 mg/kg (2.29 ± 0.22) and 10 mg/kg (2.38 ± 0.24) and with IV rtPA at 10 mg/kg (2.00 ± 0.00). In general, the rats were alert and responsive to external stimuli and were eating and drinking. They displayed body torsion and right side weakness but, in most cases, circling was not present. In contrast, rats exposed to 30 mg/kg rt-PA IA (4.30 ± 0.66) and 30 mg/kg rt-PA IV (3.60 ± 0.40) showed more severe neurological dysfunction. Multivariate analysis showed a significant effect of dose and route on the Modified Bederson Score (Table 4).

## Discussion

We tested the safety of IA versus IV rt-PA administration in a rat model of MCAo in an extended ischemic time window. The main finding of the study was that rt-PA administered by IA infusion after 6 hours of MCAo had significantly more intracerebral bleeding than did the same dosages administered IV. Indeed, even 1 mg/kg rt-PA IA caused more severe bleeding than 10 times that dose IV (10 mg/kg). The increased bleeding did not translate into a significant increase in infarct volume. The highest rt-PA dosage by both the IA and IV routes resulted in increased neurological dysfunction.

The increased bleeding with IA administration probably relates to higher concentrations of rt-PA in the local milieu. Also, rt-PA is metabolized rapidly by the liver resulting in a biological plasma half life in the rat of only 1-2 minutes [29], further exaggerating the difference in concentration after IV versus IA administration.

Cerebral ischemia predisposes the vasculature and surrounding parenchyma to hemorrhage upon vascular recanalization [30] and the vulnerability of the downstream brain parenchyma increases with longer periods of ischemia. Several pathological mechanisms underlie the ICH caused by rt-PA including, activation of MMP-9, interaction with NMDA receptors, endogenous proteolytic activity, injury to the endothelial junctional proteins contributing to the blood-brain barrier dysfunction, and activation of other proteolytic enzymes such as plasmin [31]. Taken together, it is not surprising that our results support the conclusion that similar dosages of rt-PA delivered IA carry greater ICH risk than when delivered IV.

The duration of the ischemic event may modify the toxicity of rt-PA. Increased bleeding did not occur with IV rt-PA doses as high as 20 mg/kg if administered within 1 hour ischemic onset [32], suggesting a therapeutic index (TI = Toxic dose ÷ Therapeutic dose) of greater than 2. Whereas doses of rt-PA administered after a 3 hour delay resulted in a dose-dependent (0.9, 9 and 18 mg/kg) increase in hemoglobin extravasation [33]. By extension, in our study, 30 mg/kg rt-PA IV resulted in significant bleeding following 6 hours of ischemia. Taken together, our results and the data of other groups supports the conclusion that bleeding is not induced by substantial doses of rt-PA following short periods (< 3 hours) of ischemia, but that the risk of bleeding increases significantly with longer intervals of occlusion.

Because of the implications of high concentrations of rt-PA when administered IA, we closely mimicked such conditions by use of a snare ligature model, which enabled us to initiate IA rt-PA immediately prior to vascular recanalization. Furthermore, as fibrin degradation products may contribute to rt-PA-associated ischemic damage [34,35], the snare ligature model allowed us to dissociate ischemic damage caused by rt-PA from that caused by fibrinolytic products.

The clinical therapeutic dose of rt-PA IV for ischemic stroke has been established at 0.9 mg/kg. The dose for rt-PA IA as a stand alone treatment is more variable and ranges from 20 to 60 mg [10]. Our data suggest that to avoid increased ICH risk by the IA route compared to IV, the dose would need to be considerably less, although such conclusions must be tempered by the understanding that our model may not entirely reflect the clinical state in human disease.

The 30 mg/kg rt-PA IA and IV dose groups were included in the study to provide an estimate of the therapeutic index and to exaggerate potential bleeding complications. In 3 of 5 rats, 30 mg/kg IA induced spontaneous bleeding in the brain stem that was not directly affected by the vascular ligation. All 3 rats had damage to the hypothalamus and two of these had seizure activity necessitating euthanasia. As the arterial perforators to the brain stem come off of the Circle of Willis prior to the branching of the MCA, significant blood levels of rt-PA would be present in these arteries following IA administration resulting in brain tissue exposure to high concentrations of rt-PA over the extended duration of administration (60 minutes). Hemorrhagic damage to the brain stem, hypothalamic damage and seizure activity did not occur in any animal dosed with 30 mg/kg rt-PA IV.

In our study, the Modified Bederson Score was used to assess the functional recovery of the rats. Our data suggests that the final behavior of the rats was significantly affected by both dose and route of rt-PA administration and the major driving force was bleeding. The infarct volume was not a major contributor to behavioral function after 6 hours of ischemia.

A limitation to this study was the number of rats (4 out of 5) euthanized in the 30 mg/kg IV group because of unresponsiveness to external stimuli for over 3 hours following completion of the rt-PA infusion. The Bleeding Score for these 4 rats were determined in brain sections after < 3 hours maturation following reflow, which may have underestimated the true extent of bleeding. However, 2 of the 4 rats received a score of 4 because of large space occupying hemorrhages. The other 2 rats had Bleeding Scores of 3 and 2 which were most likely accurate despite the shorter maturation time. This suggests that the premature termination probably had little effect on the Bleeding Score results.

The number of rats prematurely terminated in the both of the 30 mg/kg rt-PA dose groups may have had an impact on the infarct volume data. Of the 10 animals in these 2 groups, 7 were euthanized within 3 hours of dosing, thereby reducing the maturation of the infarct following reflow to less than 3 hours as opposed to ~18 hours. In addition, one rat dosed 30 mg/kg rt-PA IA died overnight. The lack of maturation of the infarct volume may have resulted in an underestimate of the true infarct volume in these rats.

The early termination of the rats in the 30 mg/kg IV group may have lead to an under estimation of the functional recovery of these rats. Two of the rats had surprisingly little bleeding in relation to behavior and thus, may have survived to scheduled termination. Subsequent experience suggests that survival would have been likely. The pronounced depressed behavior of these rats post dosing suggests that rt-PA may have severe intrinsic CNS depression activity at high doses.

## Conclusion

The overall conclusion of the study was that rt-PA given by IA infusion following extended ischemic insults causes greater intracerebral bleeding than when similar doses are administered IV. Indeed, IA dosages 10-fold less than administered IV may still pose a greater ICH risk. Because the model system we used was able to dissociate damage caused by rt-PA from ischemic damage and damage that may be caused by fibrinolytic degradation products, the bleeding observed in our study was likely an intrinsic property of rt-PA. Our data highlights the need for safer pharmacological thrombolytic agents especially when administered after extended ischemic durations.

## Competing interests

R. Christian Crumrine, G. McLeod Taylor, Philip Scuderi, Stephen Petteway, Jr., and Vikram Arora are employed by Grifols Therapeutics, Inc., Research Triangle Park, NC. Constantinos P. Tsipis, Joseph C. LaManna and Victor J. Marder are paid consultants for Grifols Therapeutics, Inc.

The rt-PA used in the study is commercially available.

## Authors' contributions

RCC performed the surgeries, was involved in the conception and planning of the experiment and drafted the manuscript. VJM participated in the design of the experiment and edited the manuscript. GMT participated in the execution of the experiment, in the data analysis and in the design of experimental devices. JCL participated in the analysis of data, consulted in the experimental design and provided intellectual input to the drafting of the manuscript. CPT was crucial to the analysis of the infarct volume and bleeding score data. PS, SRP and VA conceived of the experiment and participated in the planning stage.

All authors read and approved the manuscript.

## Supplementary Material

Additional file 1 **Figure S1**. Infarct Volume. Shows the extent of the infarction produced by occlusion of the MCA near the near the inferior cerebral vein (9 sequential TTC stained 2 mm fresh sections). **Figure S2**. Construction of the IA infusion Catheter. Shows the IA catheter components and the assembled catheter. **Figure S3**. Temporal progression of infarct volume. Shows the time course of infarct development in this model. **Figure S4**. Surgical Sham Animals (whole brain and TTC stained photographs). **Figure S5**. EC-ICA Sham Animals (whole brain and TTC stained photographs). **Figure S6**. Reflow as indicated by latex infusion. Click here for file
