# Supplementary material for: Intra-arterial administration of recombinant tissue-type plasminogen activator (rt-PA) causes more intracranial bleeding than does intravenous rt-PA in a transient rat middle cerebral artery occlusion model
Source: Exp Transl Stroke Med. 2011 Sep 20;3:10. doi: 10.1186/2040-7378-3-10 (PMC3184064; doi:10.1186/2040-7378-3-10)
Supplement: Additional file 1 — Figure S1. Infarct Volume. Shows the extent of the infarction produced by occlusion of the MCA near the near the inferior cerebral vein (9 sequential TTC stained 2 mm fresh sections). Figure S2. Construction of the IA infusion Catheter. Shows the IA catheter components and the assembled catheter. Figure S3. Temporal progression of infarct volume. Shows the time course of infarct development in this model. Figure S4. Surgical Sham Animals (whole brain and TTC stained photographs). Figure S5. EC-ICA Sham Animals (whole brain and TTC stained photographs). Figure S6. Reflow as indicated by latex infusion. [file 2040-7378-3-10-S1.PDF]

Intra-arterial Administration of Recombinant Tissue-Type Plasminogen Activator (rt-PA) Causes More Intracranial Bleeding than Does Intravenous rt-PA in a Transient Rat Middle Cerebral Artery Occlusion Model

R. Christian Crumrine, PhD[1], Victor J. Marder, MD[2] , G. McLeod Taylor, DVM[1], Joseph C. LaManna, PhD[3], Constantinos P. Tsipis[3],, Stephen Petteway, Jr., PhD[1], Philip Scuderi, PhD[1] and Vikram Arora, PhD[1]

[1]Research and Pre-Clinical Development, Grifols, Inc., Research Triangle Park, North Carolina, [2]David Geffen School of Medicine at UCLA, Los Angeles, California, [3]Department of Physiology and Biophysics, Case Western Reserve University, Cleveland, Ohio;.

Figure S1. Infarct Volume

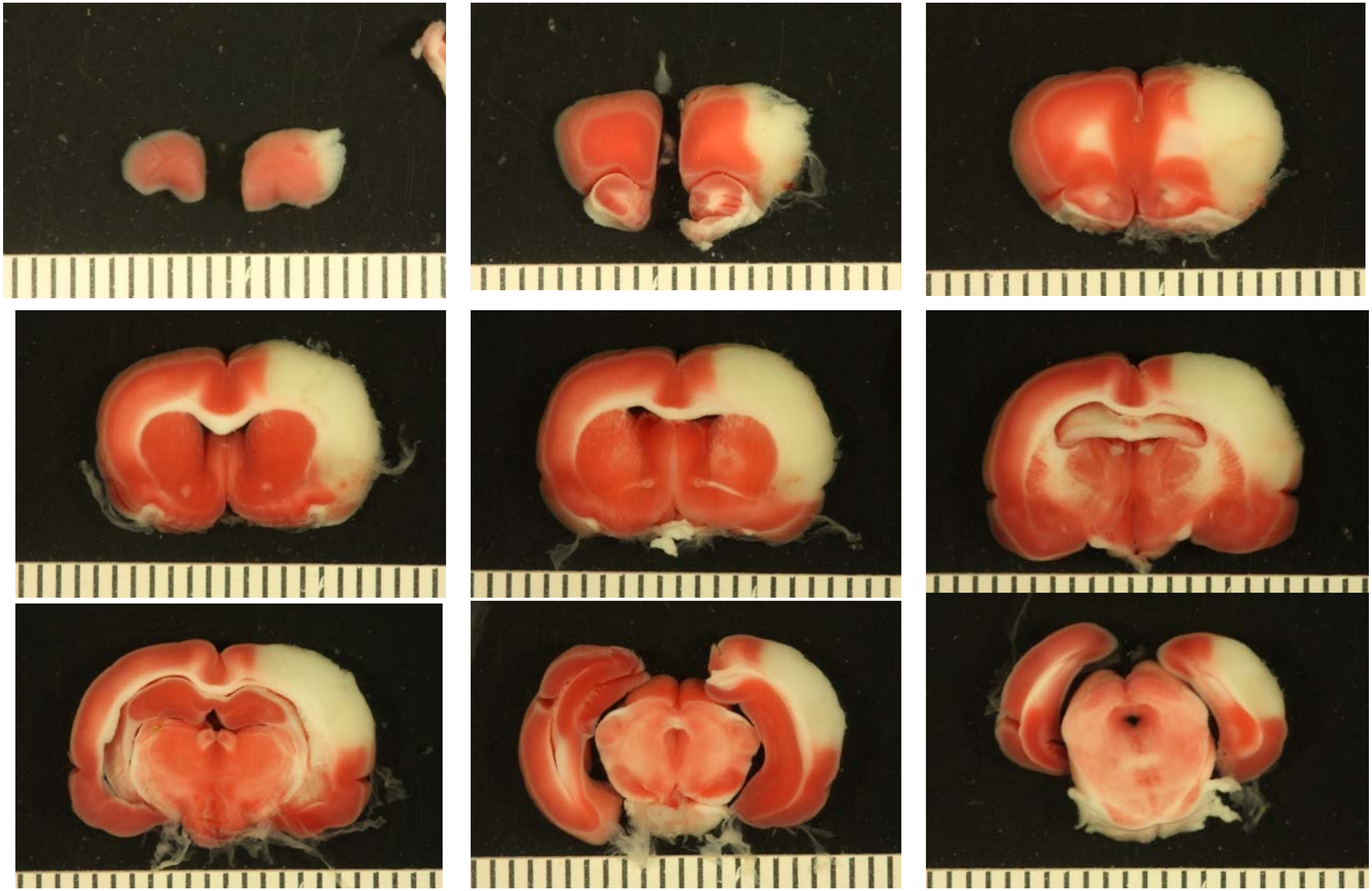

Extent of Infarct Volume. Sequential 2 mm coronal brain sections were floated in a Petri dish containing TTC at 37º C for ~15 min in the dark. The Petri dish was placed in an ice bath and the brain sections were photographed with a ruled standard. Note lack of involvement of the basal ganglia.

Figure S2. Construction of the IA infusion Catheter

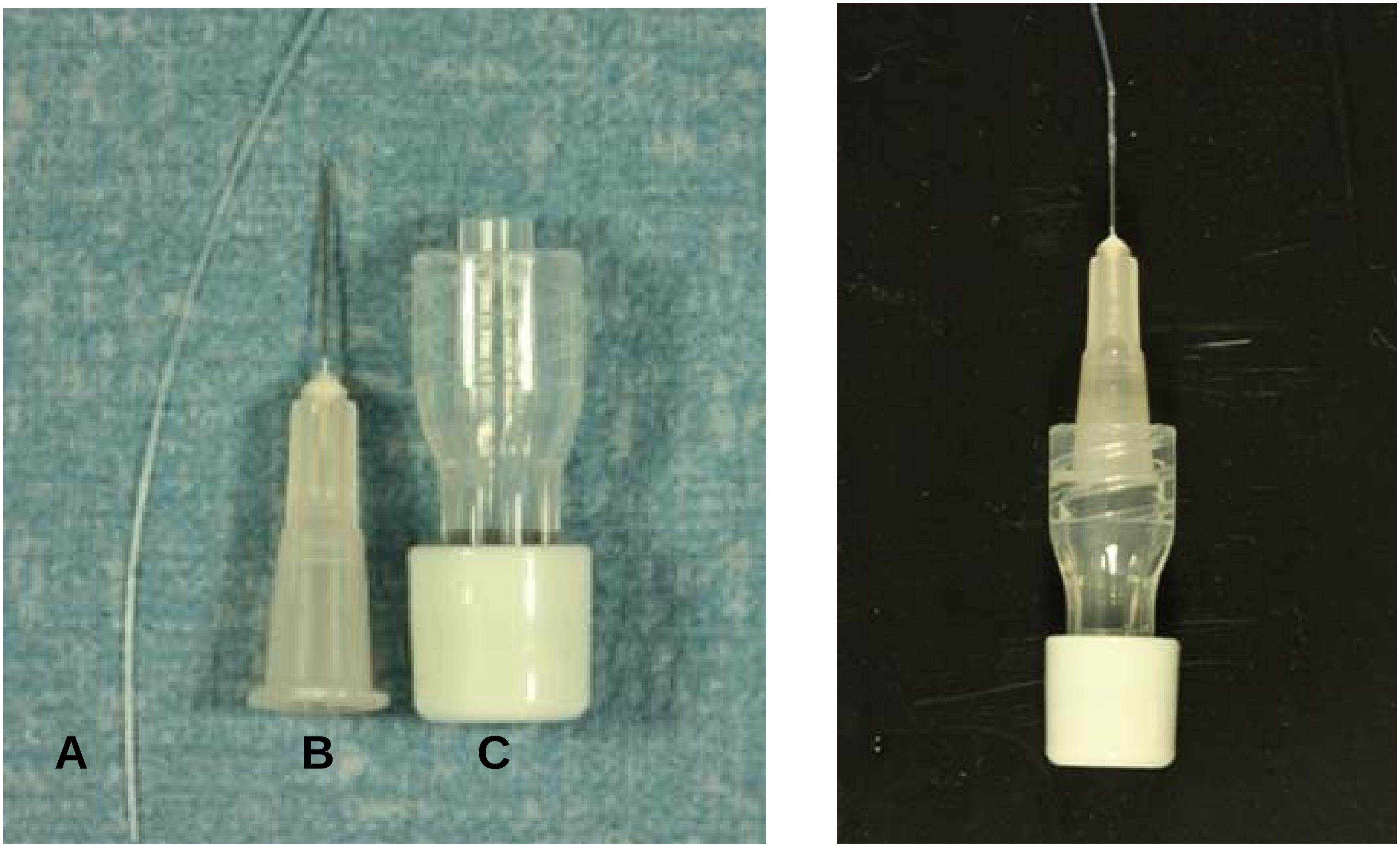

The IA infusion catheter was constructed from (left panel): A) PTFE sub-lite wall tubing (SUBL-160, OD/ID 0.41/0.25 mm), B) 30 gauge syringe needle and C) an injection port. The right panel shows the completed catheter.

Figure S3. Temporal infarct volume progression (Unpublished historical Data)

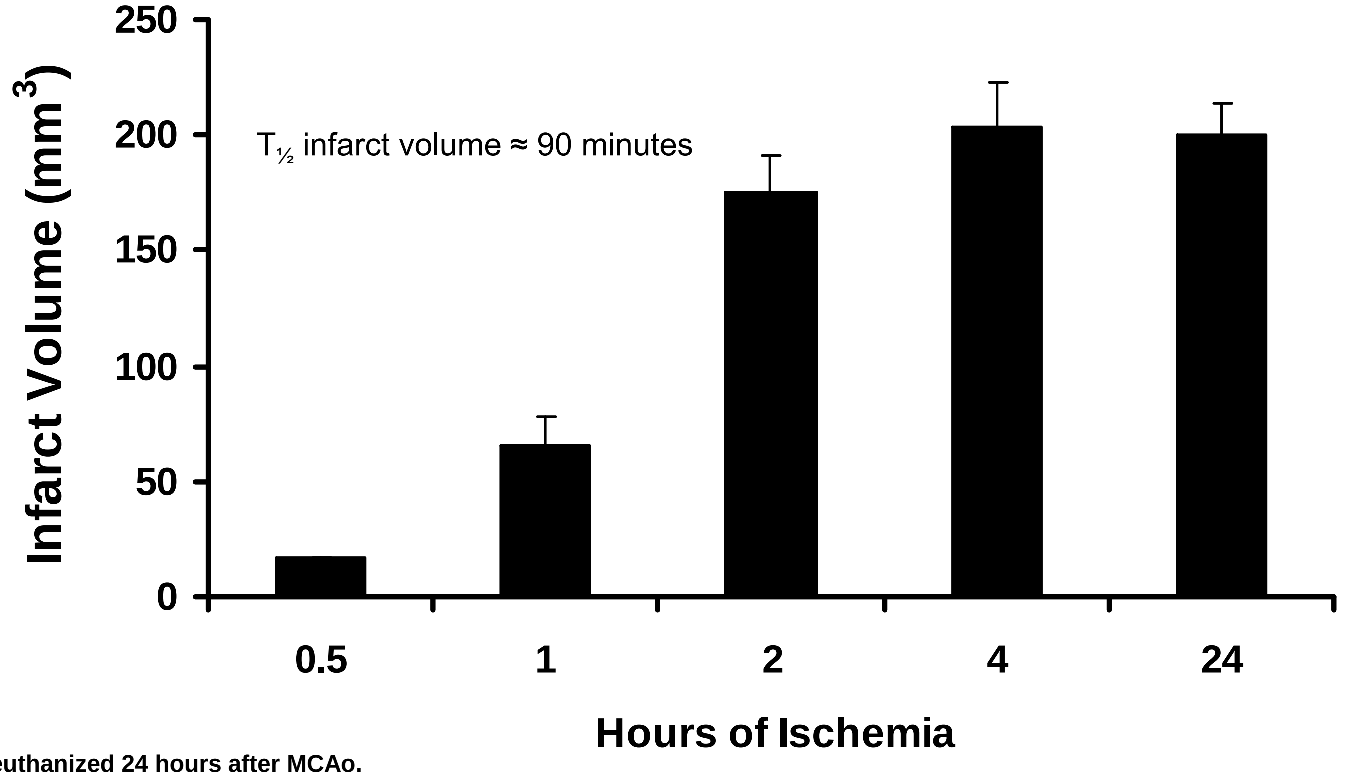

All rats were euthanized 24 hours after MCAo.
Values are the mean ± SEM (n=3, 4, 4, 4, 5, respectively)

The data for the temporal progression of infarct volume was generated by the authors (RCC and JCL) at Case Western Reserve University (CWRU) in 1987. The animal use protocol was reviewed and approved by the CWRU IACUC before beginning the study. The data are not directly comparable to the infarct data presented in the safety study because of the location of the occlusion (proximal to the lenticulostiate arteries) and the method of infarct volume determination (H&E staining of 20 μm frozen sections at 300 μm intervals).

Temporal progression of infarct damage in this model indicates that an ischemic duration of 4 hours (203 ± 19.3 mm^3) results in a mature infarct volume similar to permanently (200.7 ± 12.9 mm^3) occluded animals. Prior to that, increasing durations of MCAo results in progressive enlargement of the infarct volume (16.7 ± 0.64, 65.6 ± 12.4, 175.2 ± 15.7 mm^3 for 0.5, 1, and 2 hours, respectively). This is similar to results described by Aronowski et al [25].

Figure S4. Surgical Sham Animals

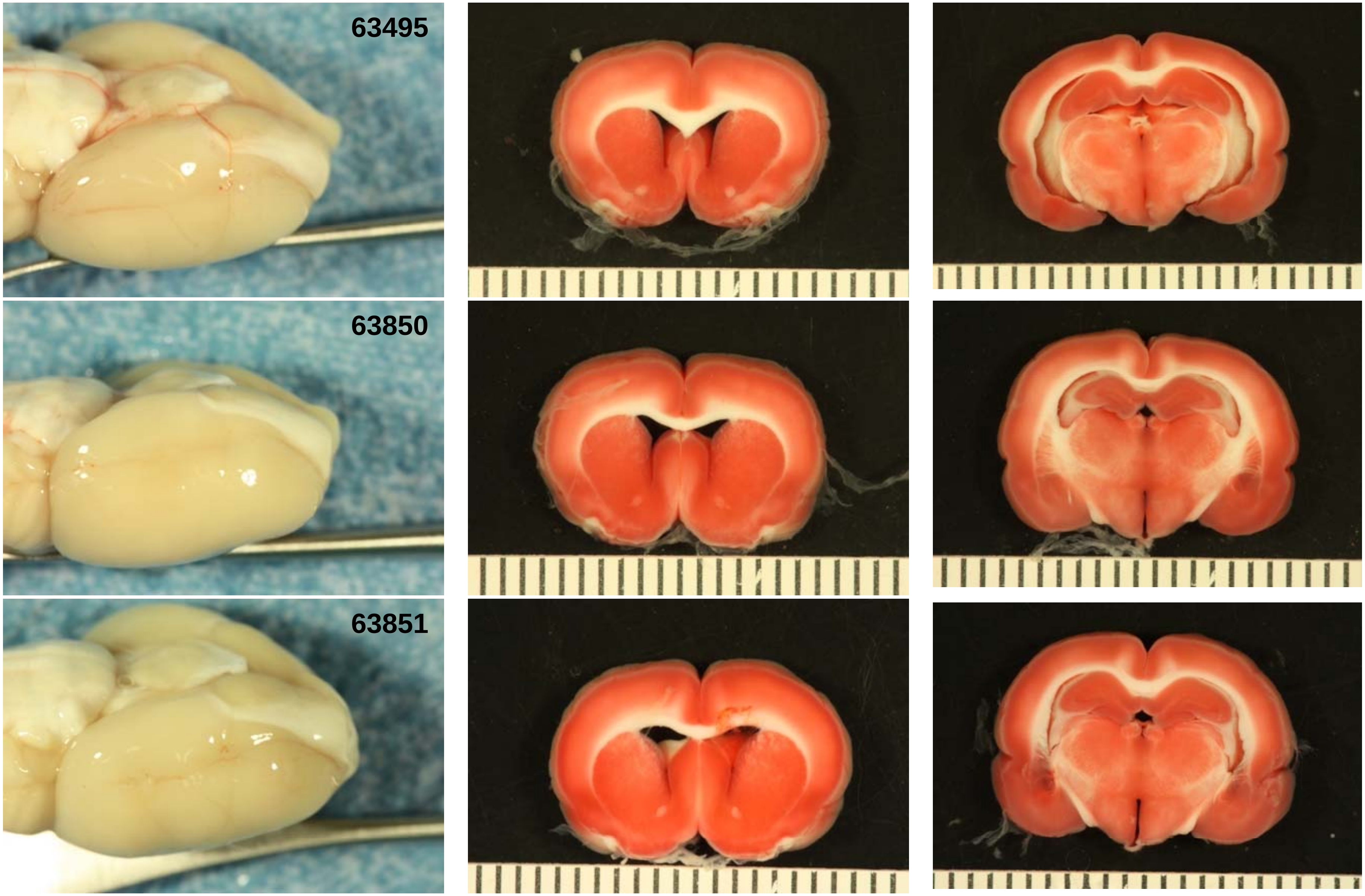

Photographs of Gross Brain and Corresponding TTC stained Coronal Sections. Rats were subjected to the MCAo surgical procedure as described in Methods. The MCA was elevated into the silastic tubing for less than 5 seconds, without occlusion, and then the snare ligature was dismantled. No ischemic damage was observed

Figure S5. ICA Sham Animals

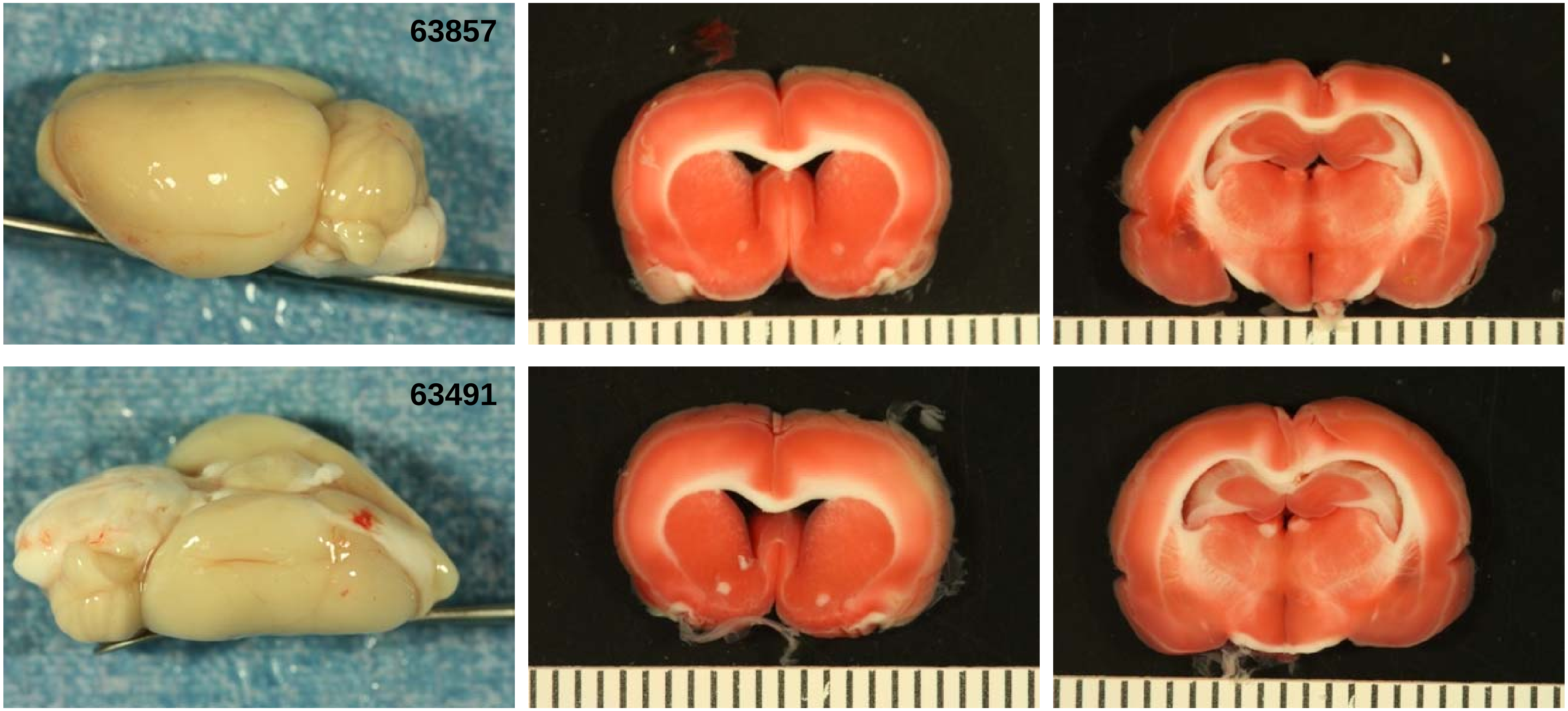

Photographs of Gross Brain and Corresponding TTC stained Coronal Sections. Rats were subjected to sham MCAo procedure as described in figure 3. Six hours later, the extra cranial internal carotid artery (EC-ICA) was cannulated and saline was infused per the IA dosing procedure. No damage to the brain was observed

Figure S6. Reflow as Indicated by Latex Infusion

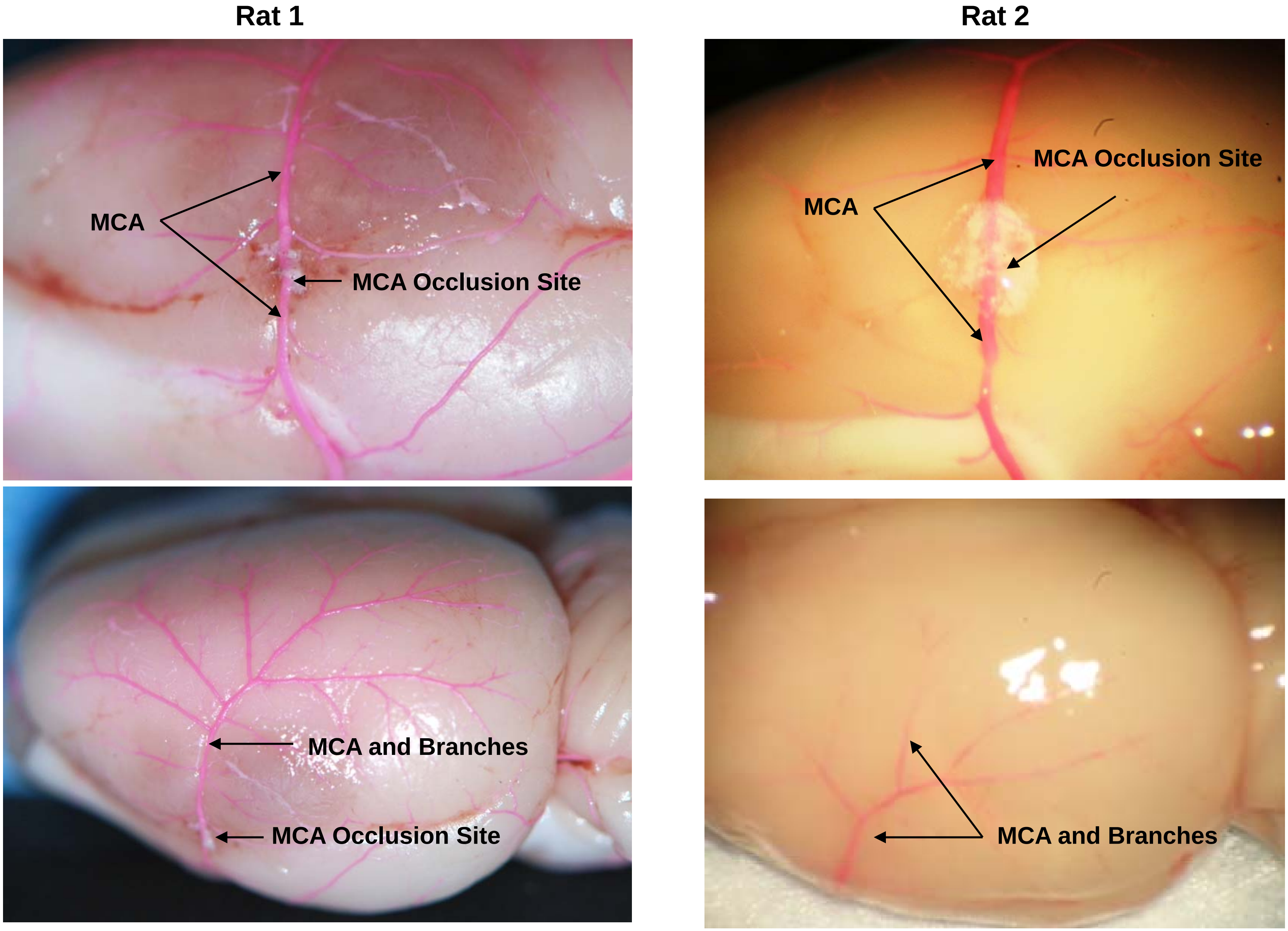

Rats were subjected to 6 hours MCAo. The EC-ICA catheter was placed and following removal of the snare ligature, the rat was euthanized and latex was infused into the EC-ICA. Filling of the MCA vascular tree indicates recanalization was achieved
